# Size‐selective harvesting drives genomic shifts in a harvested population

**DOI:** 10.1111/jfb.15901

**Published:** 2024-08-08

**Authors:** Daniel E. Sadler, Tiina Sävilammi, Stephan N. van Dijk, Phillip C. Watts, Silva Uusi‐Heikkilä

**Affiliations:** ^1^ Department of Biological and Environmental Science University of Jyväskylä Jyväskylä Finland; ^2^ Department of Biology University of Vermont Burlington Vermont USA

**Keywords:** fisheries‐induced evolution, genetic variation, population genomics, size‐selection, whole‐genome sequencing

## Abstract

Overfishing not only drastically reduces the number of fish in an exploited population but is also often selective for body size by removing the largest individuals from a population. Here, we study experimentally the evolutionary effects of size‐selective harvesting using whole‐genome sequencing on a model organism, the zebrafish (*Danio rerio*). We demonstrate genomic shifts in the populations exposed to size‐selective harvesting for five generations and show reduced genetic diversity in all harvested lines, including the control line (non‐size‐selected). We also determine differences in groups of genes related to certain gene ontology annotations between size‐selectively harvested lines, with enrichment in nervous system related genes in the large‐selected lines. Our results illuminate the biological processes underlying fisheries‐induced genetic changes and hence contribute toward the understanding of the changes potentially associated with the vulnerability of an exploited population to future stressors.

## 1 INTRODUCTION

Harvesting of animals frequently exceeds natural mortality rates and can cause drastic demographic changes in a population (Festa‐Bianchet et al., 2011; Jørgensen et al., 2007). Overfishing is a particularly severe example of harvesting, as adult fish are removed from populations at an unprecedented rate, often exceeding fishing mortality rates of 75% (Lewin et al., 2006). Such rapid population decline can lead to loss of genetic (Marty et al., 2015; Pinsky & Palumbi, 2014; Therkildsen et al., 2019) and phenotypic (Olsen et al., 2009) variation. Alongside decreased population sizes, many fisheries also exert directional selection on body size, often removing the largest individuals from the population (Jørgensen et al., 2007; Law, 2007; Lewin et al., 2006). Such directional selection on body size can drive changes in phenotypic traits, including faster juvenile growth rate, earlier maturation, and altered behavior (Mollet et al., 2007; Olsen et al., 2004; Reid et al., 2023; Uusi‐Heikkilä et al., 2015; van Wijk et al., 2013). Though demographic and phenotypic changes are often clearly visible over time in an exploited population, size‐selective harvesting can also cause genetic changes underlying the phenotypic ones (Therkildsen et al., 2019; Uusi‐Heikkilä et al., 2015, 2017; van Wijk et al., 2013). Identifying genetic changes caused by fishing is important as they are slow to reverse, if indeed can be reversed at all (Conover et al., 2009; Lacy, 1987). Now that we are in the age of genomics, we can look further into the mechanisms and associated functions induced by size‐selective fisheries using next‐generation sequencing technology.

Size‐selective fishing can result in decreased genetic diversity (Marty et al., 2015; Pinsky & Palumbi, 2014; Poulsen et al., 2006; Therkildsen et al., 2010). A reduction in genetic diversity can be problematic in rapidly changing environments because it can lead to a loss of adaptive potential (Allendorf et al., 2008; Fisher, 1958). Evidence for erosion of genetic diversity in exploited fish stocks is accumulating, showing increases in inbreeding coefficient or reductions in effective population size (*N*_*e*_) (Hauser et al., 2002; Hoarau et al., 2005; Hare et al., 2011; see Pinsky & Palumbi, 2014 for meta‐analysis). Despite growing evidence of decreased genetic diversity, some studies show no declines in genetic diversity caused by fishing (Hutchinson et al., 2003; Poulsen et al., 2006; Ruzzante et al., 2001; Therkildsen et al., 2010; reviewed by Sadler et al., 2023), potentially because many fish populations are so large that even collapsed populations are resistant to the loss of genetic diversity (Andersen & Brander, 2009; Beverton, 1990).

Alongside demographic changes (i.e., decreased density), size‐selective fisheries also exert directional selection, which could magnify the loss of genetic diversity compared to population loss per se (Frankham, 2012). Experimental studies have demonstrated significant genetic changes in artificially harvested fish populations after only three (van Wijk et al., 2013), four (Therkildsen et al., 2019), or five generations (Uusi‐Heikkilä et al., 2015, 2017) of size‐selective harvesting. The cause of these changes can be challenging to manifest in natural populations because fishing occurs in a constantly changing environment where biotic and abiotic conditions can create simultaneous selection pressures to fish populations. In an experimental system, confounding factors such as these environmental changes can be removed to isolate the effects of size‐selection alone; however, even in experimental studies these changes can be difficult to replicate. Therkildsen et al. (2019) demonstrated genomic changes in different size‐selected experimental populations of Atlantic silverside (*Menidia menidia* L.). Each experimental population had two replicates, which showed both parallel and idiosyncratic effects on the genome but no phenotypic responses to the exact same selection pressure. These results highlight how different evolutionary trajectories from different islands of genomic architecture can lead to the same phenotypes and potentially complicate the predictions of the outcomes of fisheries‐induced evolution due to the added stochasticity of genomic changes. It is therefore fundamental that we assess whether this stochasticity is repeatable across other systems, if so then it will make genomic shifts more difficult to comprehend for fisheries management.

Gene ontology describes functions of genes and gene products (Ashburner et al., 2000), and thus it can help to interpret fisheries‐induced genetic changes. Moreover, gene ontology can be used to link genetic changes to potential phenotypic changes, including growth, metabolic rate, and behavior. However, this is possible only when using a model organism such as the zebrafish, *Danio rerio* (Hamilton, 1822), which has a high‐quality, high‐resolution reference genome (Howe et al., 2013). As such, although an earlier size‐selective harvesting experiment has described large‐scale genomic changes (Therkildsen et al., 2019), no study has utilized this framework within a model system. By assessing gene ontology, we are able to estimate the underlying functionality of hitchhiker genes that could be associated with the selection of body size that are unclear when assessing phenotypic changes alone. Thus, we are able to expand on previous studies and identify key differences in gene functions and molecular processes, further allowing us to predict the pathways associated with enriched genes.

In the present study we assessed how *D*. *rerio* that had experienced size selection differed in their genomic architecture after five generations of simulated harvesting. Selection lines consisted of (1) small‐selected (simulating typical fisheries selection), (2) large‐selected (to understand the full range of responses caused by size selection), and (3) random‐selected (no size selection). We assessed the large‐scale genomic changes caused by intensive size‐selective harvesting at the whole‐genome level. We hypothesized that (1) size‐selective harvesting will cause a shift in genomic architecture over a contemporary time scale, (2) directional selection (large‐ and small‐selected lines) will have a greater loss of genetic diversity and corresponding shift in genomic architecture after five generations of harvesting than population reduction alone (random selection), and (3) associated gene ontology terms will relate to growth‐associated functions and be more enriched in populations exposed to directional selection.

## 2 MATERIALS AND METHODS

### 2.1 Experimental design

We used wild‐caught *D*. *rerio* originating from West Bengal, India, as the *F*_0_‐generation (founder population) in this size‐selective harvesting experiment (Uusi‐Heikkilä et al., 2010). In this experiment, we used three selection lines, each with 450 individuals per line replicate having experienced 75% fishing mortality rate (Uusi‐Heikkilä et al., 2015): (1) small‐selected (75% of the smallest fish were harvested), (2) large‐selected (75% of the largest fish were harvested), and (3) random‐selected (75% randomly chosen fish were harvested, acting as the control no size‐selection treatment) over five generations. Fish were harvested when 50% of the fish reached maturity. Each selection line contained two replicate populations, and is henceforth called SS1 (small‐selected), SS2, LS1 (large‐selected), LS2, RS1 (random‐selected), and RS2. Full details of the experimental design can be found in Uusi‐Heikkilä et al. (2015).

### 2.2 Ethics statement

All methods were performed in accordance with the relevant guidelines and regulations. All experimental protocols were approved by the Finnish Project Authorisation Board, license number: ESAVI/24875/2018.

### 2.3 DNA extraction and sequencing

We extracted DNA altogether from 267 individuals (Table S1). We used a modified salt extraction protocol to extract the genomic DNA (Aljanabi & Martinez, 1997). Library construction and sequencing were conducted at Novogene Biotech Co. (Beijing, China). The random fragmentation of the genomic DNA was skipped because the DNA was fragmented during the storage period. The DNA fragments were end‐polished, A‐tailed, and ligated with the full‐length Illumina adapters, followed by further PCR amplification with P5 and indexed P7 oligos. The PCR products were purified with an AMPure XP system. The size distribution of the finished libraries was inspected by an Agilent 2100 bioanalyser (Agilent Technologies, CA, USA) and quantified using real‐time PCR. The quality‐controlled libraries were pooled and sequenced using the Illumina NovaSeq 6000 platform to sequence 150‐base paired end reads producing the average of 7.7 gigabases of data per sample.

### 2.4 Data assembly

We filtered the raw sequence for adapters and poor‐quality bases using fastp v. 0.20.0 (parameters‐*g‐q 5‐u 50‐n 15‐l 30–overlap_diff_limit 1–overlap_diff_percent_limit 10*). During trimming, we removed bases from read ends when they represented adapter sequence, or when they were of low quality. All assembly tools utilized htslib 1.10. We mapped the trimmed sequences against reference genome with bwa mem v. 1.10. We used the primary assembly sequences of *D*. *rerio* GRCz11 obtained from Ensembl (Howe et al., 2021) as a reference. We excluded the potential PCR duplicate reads using samtools markdup 1.10. Single‐nucleotide polymorphism (SNP) calling was done using bcftools mpileup v. 1.10 (with parameter‐a “FORMAT/DP”). To extract the per‐sample sequencing depths, we used bcftools call v. 1.10 (parameters–m‐f GQ). The SNPs were filtered and average coverages of >3 and <10 bases included using bcftools filter v. 1.10 (−i Qual>20 && AVG [FMT/DP] >3 && AVG [FMT/DP] <10). Finally, we required that each SNP was genotyped in >70% of the samples and had minor allele frequency (MAF) >0.05. We further excluded loci with more than two alleles or alleles other than 0 or 1. The sequencing resulted in the median of 7.5 and 7.3 gigabases of raw and filtered sequence data per sample, respectively. We excluded 14 individuals from further analysis due to low mapping back rate and low coverage. After being filtered, 3.5 million SNPs were called.

### 2.5 SNP annotation

The functional associations between *D*. *rerio* genes and each SNP were predicted using snpEff software v. 5.0 (default parameters; Cingolani et al., 2012) and the GRCz11 genome annotation of the *D*. *rerio*. The gene annotation with the most severe effect was selected for each SNP. If the SNP did not associate with any gene, it was annotated as intergenic.

### 2.6 Assessing genomic shifts

To visualize the genomic differences among the selection lines and the founder population, we used 1 million randomly sampled SNPs in a principal component analysis. To this end, we imputed the missing genotypes with the median allele for each SNP. We required that the subsets had MAF >0.05. Based on Cattell's graphical rule and the broken stick method (Figure S1), we determined the principal components that contained the relevant relatedness information and population structure for further analyses utilizing principal components with PCAdapt v. 4.3.3 (Luu et al., 2017). Moreover, to disentangle the effect of genetic drift from selection on genetic differentiation (*F*_ST_), we compared the *F*_ST_ between ancestral and *F*_6_ replicate populations to a simulated *F*_ST_ estimate under a drift‐only scenario. The drift‐only *F*_ST_ was obtained by simulating six generations of individuals and quantifying the level of *F*_ST_ in the absence of selection. We imputed missing genotypes using Beagle software v. 5.4 and then used SLiM 4 software (Haller & Messer, 2023) for simulations. We set recombination rate to 1e‐8 and population size to 125 individuals. We repeated the simulations for 50 times per each chromosome and averaged over the *F*_ST_ distribution to get an estimate.

### 2.7 Parallel effects

To quantify the level of parallel differentiation between the replicates of small‐ and large‐selected lines, when compared against the random‐selected lines, we used diffstat statistic (Turner et al., 2011). We extracted the minimum absolute difference between the allele frequencies of each comparison between an *F*_6_ selection line replicate population and the ancestral population to explore if the differences were consistent (i.e., the direction of allele frequency change was the same among the selection‐line replicates) among both comparisons. We set the diffstat of the loci with non‐consistent allele frequency changes to zero. CIs were estimated parametrically using single‐sample *t*‐test function.

### 2.8 Differentiation‐based outlier detection

To identify diverged loci among all selection‐line replicates and the founder population, we used two different outlier approaches. First, to detect variation that was causing divergence between the individuals regardless of the selection‐line assignment or relatedness between individuals, we used PCAdapt (Luu et al., 2017) with *K* based on Cattells rule (Figure S1; *K* indicates principal component level). We used the component‐wise analysis, which uses principal component loadings as a test statistic as the two most explanatory components clearly separate the selection lines and time (*F*_0_–*F*_6_). The resulting *p*‐values were corrected for multiple comparisons using the Benjamini–Hochberg approach (Benjamini & Hochberg, 1995). Second, to screen the genomes for signatures of response to harvesting while simultaneously controlling for random effects due to factors such as variability in population structure, relatedness of the individuals, and selection‐line replicates, we used latent factor mixed model (LFMM), implemented in the lfmm R package v. 1.0 (Frichot et al., 2013), which controls for random effects, including drift, and distinguishes them from selection. The model includes the SNP matrix as a response and selection line as a predictor, with the selection line coded as 0 or 1 per comparison. Additionally, latent factors, which are inferred from the data using the software, are used to correct the model for confounding effects of unobserved variables. Based on principal component significance, which may be used to detect the number of latent factors to consider, we used *K* = 3, which corresponds to the number of latent factors evaluated (i.e., selection lines) and is the number of expected genetic clusters. We required the final set of candidate outlier loci to be identified using both the outlier analyses (PCAdapt and LFMM).

### 2.9 Gene ontology analysis

We sought gene ontology enrichments among the genes associated with candidate outlier SNPs related to the differentiation between large‐ and random‐selected lines, between small‐ and random‐selected lines, and between line replicates. We compared the lists of all genes associated with the final sets of candidate outlier SNPs (large vs. random and small vs. random) to a background list of all genes associated with SNPs. We used the standard hypergeometric statistics, as implemented in the gene ontology enrichment analysis and visualization tool (GOrilla; Eden et al., 2009).

### 2.10 Genetic diversity

Effective population size (*N*_e_) was calculated for each population using the linkage disequilibrium method (Waples & Do, 2010) with NeEstimator v. 2.1 using random mating (Do et al., 2014). NeEstimator was used on a subset of 100,000 SNPs. VCFtools (v0.1.16) was used to calculate nucleotide diversity (π) and heterozygosity across all SNPs. The abundance of polymorphic loci (in percentages) was also calculated using a subset of 100,000 SNPs. See expanded methodology in Supplementary Materials for further details.

## 3 RESULTS

### 3.1 SNP annotation

Most of the SNPs were found in the introns and intergenic regions followed by up‐ and downstream regions of genes (Figure S2). Synonymous SNPs and 5′ UTR variants showed higher divergence (Figure S2), suggesting that directional selection has targeted functionally relevant SNPs regulating gene expression levels.

### 3.2 Genetic changes caused by size‐selective harvesting

**FIGURE 1:**
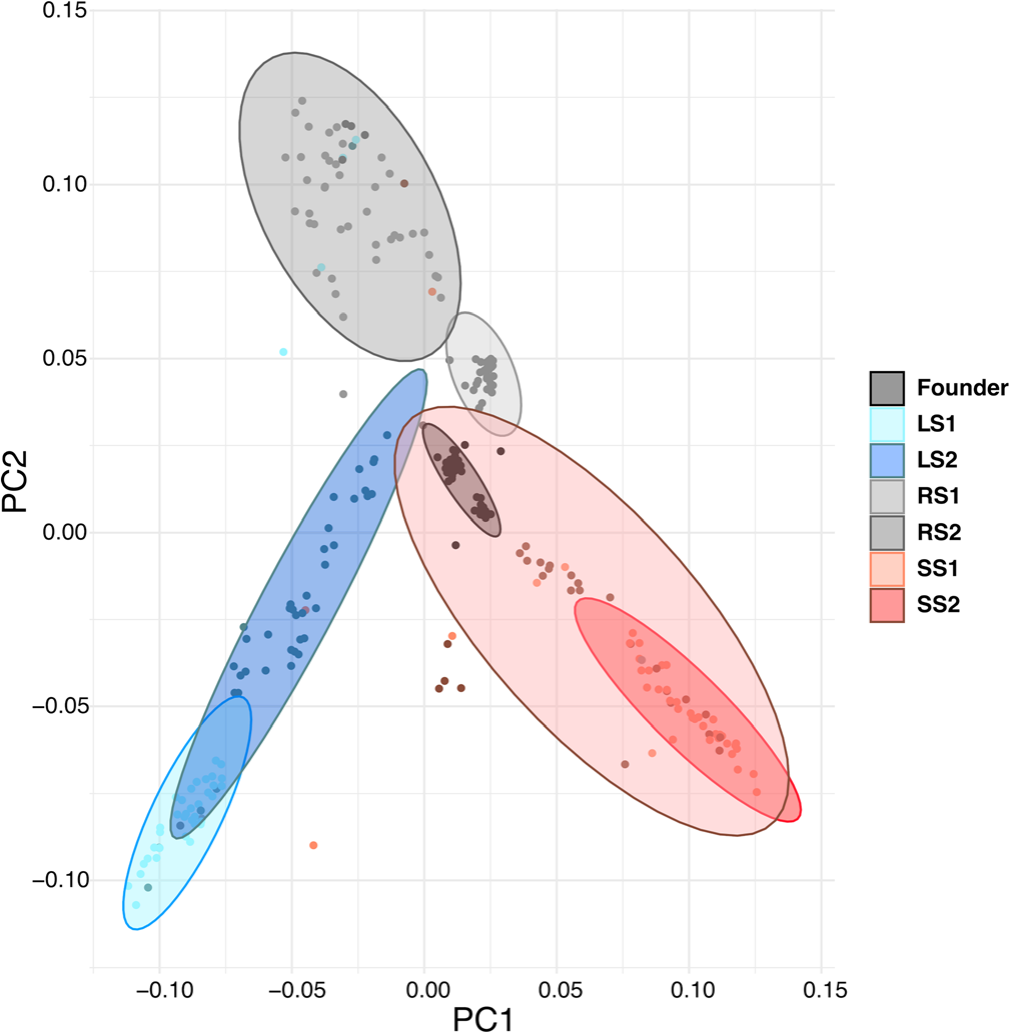
Principal component analysis based on random subset of single nucleotide polymorphisms (SNPs) among small‐selected replicates (SS1, SS2), large‐selected replicates (LS1, LS2), random‐selected replicates (RS1, RS2), and founder population. PC1 and PC2 explain 3.5% and 2.5% of the variation, respectively. Points indicate individuals. Ellipses are 95% CIs around the mean eigenvalue of each replicate and highlight selection‐line replicates and the founder population.

Fish experiencing directional selection (small‐ and large‐selected) and random selection formed distinct clusters in the principal component analysis, indicating a genomic differentiation between selection regimes. Selection lines were significantly separated from each other in both PC1 (*F*_6,260_ = 199.38, *p* < 0.001) and PC2 (*F*_6,260_ = 95.62, *p* < 0.001). PC1 separated the small‐selected line from the other two selection lines (large‐ and random‐selected; Tukey's honest significance test [HSD], *p* < 0.001; Figure 1; Table S2), whereas PC2 separated the random‐selected line from the founder population (*F*_0_‐generation; Tukey's HSD, *p* < 0.001; Figure 1; Table S3) from the lines experiencing directional selection. Furthermore, lines experiencing directional selection diverged more from the founder population than random‐selected line as evidenced by the diffstat values (Figure S3). Moreover, the lines experiencing directional selection more diverged from each other than either line from the random‐selected line. The simulated *F*_ST_ values were significantly lower (*F*_6,121_ = 281.60, *p* < 0.001) than the *F*_ST_ values of the actual *F*_6_ selection lines, demonstrating the genomic difference among the selection lines after five generations of harvesting. This suggests that the genomic differences among the selection lines were caused not only by size‐selective harvesting and but also by genetic drift (Figure S4; Table S4).

### 3.3 Outlier detection

**FIGURE 2:**
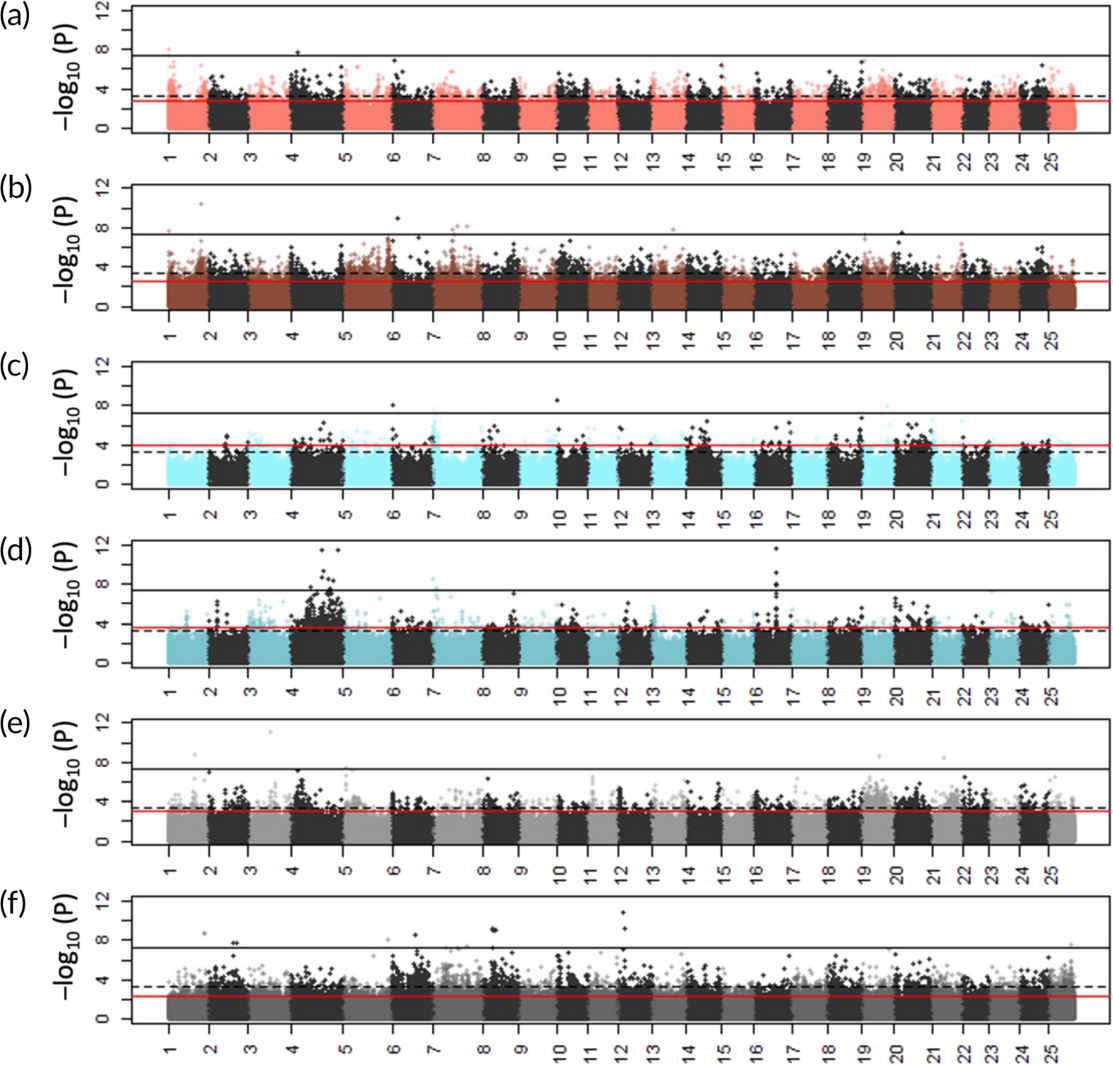
Genome‐wide distribution of *p*‐values, false discovery rate (FDR)‐corrected and −log_10_‐transformed, for candidate outlier loci of comparisons between the selection‐line replicates against the founder population, identified in the latent factor mixed model (LFMM) analysis across the 25 chromosomes. (a, b) Small‐selected replicates (SS1 and SS2); (c, d) large‐selected replicates (LS1 and LS2); and (e, f) random‐selected replicates (RS1 and RS2).

PCAdapt approach resulted in 93,397 and 15,635 candidate outlier SNPs for the first two principal components, respectively (false discovery rate [FDR] <0.01; Figure 2). Of those, 2990 were common to both PC1 and PC2 outlier sets. When small‐ and random‐selected lines were compared, 13,022 candidate outlier SNPs (associated with 5092 genes) were identified in both the LFMM and PCAdapt analysis. When large‐ and random‐selected lines were compared, 7108 candidate outliers were significant (associated with 2908 genes). When populations after five generations of harvesting were compared to the founder population, 16,090 candidate outlier SNPs were identified in the small‐selected lines, 6972 between the large‐selected lines, and 5838 between the random‐selected lines.

### 3.4 Genetic diversity

Genetic diversity, measured as % nucleotide polymorphism, effective population size (*N*_e_), and expected heterozygosity (*H*_exp_), was higher in the founder population compared to small‐, large‐, and random‐selected lines, suggesting that the 75% harvesting regime reduced genetic diversity (Table S5; Figure S4). However, this pattern was not evident in nucleotide diversity (Table S5), but we did show considerable divergence in a subset of genes and some functional regions (Figure S2). Despite differences in selection regimes, genetic diversity measures did not differ between the selection lines (Table S5).

### 3.5 Gene ontology

**FIGURE 3:**
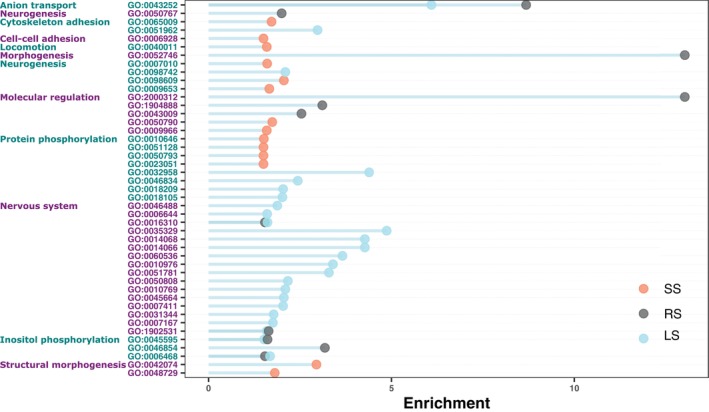
Outlier gene ontology (GO) terms with significant enrichment (false discovery rate [FDR] <0.05) from the size‐selected lines: large‐selected (LS), small‐selected (SS), and random‐selected (RS) clustered by descriptor terms. The clusters of GO terms are indicated with color changes in the term list based on term category.

We found that 212, 76, and 65 gene ontology terms were significantly enriched among the candidate outlier associated genes in small‐, large‐, and random‐selected lines, respectively (Figure S5). After being filtered for genes with the highest enrichment (>1), 25, 14, and 11 genes were enriched in small‐, large‐, and random selected lines, respectively (Figure 3). Large‐selected fish had several enriched terms related to phosphorylation and nervous system development, whereas small‐selected fish showed enrichment in structural morphogenesis and locomotion (Figure 3). Despite having fewer enriched terms, the random‐selected line had the highest individual enrichments associated with anion transport, morphogenesis, and molecular regulation (Figure 3).

## 4 DISCUSSION

Size‐selective harvesting can lead to substantial shifts in genomic architecture (Therkildsen et al., 2019; Uusi‐Heikkilä et al., 2017). Furthermore, size‐selective harvesting has been shown to reduce genetic diversity (Marty et al., 2015; Pinsky & Palumbi, 2014; Poulsen et al., 2006; Therkildsen et al., 2010). Here, we show that experimental size‐selective harvesting led to large‐scale genomic shifts, which were responsible for different gene enrichments among selection lines. Indeed, we discovered a large amount of gene ontology terms associated with nervous system in large‐selected fish. Genetic diversity decreased during the experiment (i.e., all selection lines had lower genetic diversity than the founder population); however, in contrast to our predictions, there were no clear differences in genetic diversity between the size‐selected (small‐ and large‐selected) and random‐selected lines (no directional selection).

Our results follow a suite of previous studies that demonstrate a reduction in genetic diversity after overharvesting (Marty et al., 2015; Pinsky & Palumbi, 2014; Poulsen et al., 2006; Therkildsen et al., 2010). Though it seems evident that genetic diversity should decrease following large reductions in population size and selective sweeps, some studies show no loss of genetic diversity following overexploitation (e.g., Pinsky et al., 2021). Indeed, we show clear loss in overall genetic diversity but no loss in nucleotide diversity, though we do show divergence in functional regions likely targeted by selection. Interestingly, here we show that genetic diversity did not differ between populations exposed to directional selection (small‐ and large‐selected) and those exposed to random selection. This is contrary to Therkildsen et al. (2019) who showed that size‐selected lines had lower genetic diversity compared to random‐selected lines after four generations of harvesting with a harvesting rate of 90%. Such contrasts in results may be expected due to differences in methodology (e.g., high vs. low coverage), life history, and ecology of studied species (*M. menidia* vs. *D*. *rerio*) (Sadler et al., 2023). Though genetic loss can be indicative of reduced adaptive potential (Allendorf et al., 2008), it is important to investigate what allele variations are being selected against and how genomic architecture changes depending on the harvesting protocol (i.e., directional selection or random selection).

Although the phenotypic effects of size‐selective harvesting (reduced body size, earlier maturation, and reduced reproductive output) have been demonstrated earlier (Conover & Munch, 2002; Uusi‐Heikkilä, 2015; van Wijk et al., 2013), it has remained less clear what is the magnitude of genetic changes caused by harvesting. Previous studies have demonstrated this by utilizing relatively a small number of genetic markers (Marty et al., 2015; Uusi‐Heikkilä et al., 2015; van Wijk et al., 2013), whereas whole‐genome approaches have been less common (but see Therkildsen et al., 2019; Pinsky et al., 2021). Here, we show that size‐selection shifts genomic architecture (Figure 1), with directional selection (large‐ and small‐selected populations) having different evolutionary trajectories compared to random selection. Directional selection can cause, over time, genetic changes through genetic hitchhiking and selective sweeps (Frankham, 2012; Stephan, 2019; Therkildsen et al., 2019). Genetic hitchhiking is a process where allele frequency is changed because the locus is linked to another gene that is under selection. This can reduce the amount of genetic variation in a population, especially near the selected site. A selective sweep, on the contrary, is a process where the frequency of beneficial alleles is increased, and in the most extreme case, the allele becomes fixed (Stephan, 2019). Here, selective sweeps could be driving differences in genomic architecture of the large‐selected, small‐selected, and random‐selected lines, though due to the polygenic nature of body size, it is difficult to pin down specific alleles. Small‐selected lines were most different from other selection lines as shown by PC1 (Figure 1); this follows the pattern of phenotypic differences previously shown among the selection lines (increased fecundity, smaller body size, and decrease in boldness) (Uusi‐Heikkilä et al., 2015). We also show that directional selection (small‐ and large‐selected line) drove genomic shifts in a different direction compared to reductions in population size alone (random‐selected line). Furthermore, the change in genomic architecture and genetic diversity means intensive harvesting could lead to severe loss in adaptive potential (Hollins et al., 2018), which may act in tandem with phenotypic changes to erode population resilience to environmental change (Morrongiello et al., 2021; Sadler et al., 2024; Wootton et al., 2021). Despite experiencing the same selective pressure, genomic changes differed between population replicates (see also Therkildsen et al., 2019), suggesting different evolutionary trajectories toward different genomic architecture. Though differences in genomic architecture occur despite experiencing the same selective pressure, the genetic variation is likely to be high enough to maintain genetic redundancy, with different genes producing the same phenotype with different pathways to the same function (Barghi et al., 2019).

Due to the extensive *D*. *rerio* reference genome annotations, we were able to produce high‐quality, accurate gene ontology associations to the outlier SNPs (Howe et al., 2013). The high‐quality reference genome allowed us to predict the functions being selected for as a result of size selection. We showed large clusters of enriched genes associated with nervous system function in large‐selected lines, which could be indicative of, for example, changes in behavior or cognition. It was demonstrated earlier that the large‐selected fish were more explorative and bolder than small‐selected individuals (Uusi‐Heikkilä et al., 2015), and they have also been shown to differ in their personalities and cognitive functions even after harvesting had been halted for generations (Roy et al., 2023; Sbragaglia et al., 2019). In contrast to the large‐selected lines, lowered activity and increased cautiousness of small‐selected individuals have been evident in other studies (Walsh et al., 2005), and this can lead to increased vulnerability to fishing gear (Alós et al., 2012; Diaz Pauli et al., 2015; Härkönen et al., 2014). Here, we may provide evidence of supporting genetic changes to behavior via nervous system developmental changes, though we can only show inference with gene ontology terms without gene expression data. Furthermore, we see more gene enrichment in lines exposed to directional selection (small‐ and large‐selection lines) than to random selection. Indeed, differentiation of gene ontology could allow us to predict which genes are being hitchhiked along with size‐related genes during size selection. Moreover, we show that the different line replicates differ in their gene ontology despite undergoing the same selective pressure (Figure S5), further supporting the theory that size selection can drive different evolutionary trajectories resulting in differing genomic architecture.

We show that harvesting, whether being directional for body size or not, decreases genetic diversity and causes divergence in genomic architecture, with directional selection favoring small fish causing greater genomic shifts than random selection. Although this may imply that size‐selective harvesting removing the largest individuals from the population causes greater genomic changes than balanced harvesting (i.e., no size selection, random selection), it is noteworthy that it did not cause greater losses in genetic diversity. However, type of selection should be considered in fisheries management, as changes in genomic architecture can potentially lead to shifts in population vulnerability through changes in functions and loss of adaptive alleles. Here, we show a detailed gene ontology from genomic data to understand associated genes that have been selected for alongside size‐related phenotypes. However, future studies should consider other approaches, such as metabolomics, to get an in‐depth picture of differentiation of pathways leading to phenotypic change. Furthermore, as we provide further evidence of divergence of genomic architecture following the same size‐selection regime (Therkildsen et al., 2019), it would be plausible to see if this is a consistent effect over multiple selection lines and whether the effects are truly stochastic. Overall, reductions in genetic diversity and changes in genomic architecture can lead to the loss of adaptive alleles in populations, making them more prone to future environmental stressors and fisheries events.

## AUTHOR CONTRIBUTIONS

Silva Uusi‐Heikkilä, Phillip C. Watts, Tiina Sävilammi, and Daniel E. Sadler conceived the ideas and designed the methodology. Daniel E. Sadler and Stephan van Dijk collected the data. Daniel E. Sadler and Tiina Sävilammi analysed the data. Daniel E. Sadler led the writing of the manuscript. All authors contributed critically to the drafts and gave their final approval for publication.

## FUNDING INFORMATION

This study was funded by Academy of Finland (grant number: 325107).

## Supporting information


**Data S1.** Supporting information.
