# Supplementary material for: Size‐selective harvesting drives genomic shifts in a harvested population
Source: J Fish Biol. 2024 Aug 8;105(6):1562–71. doi: 10.1111/jfb.15901 (PMC11650958; doi:10.1111/jfb.15901)
Supplement: Supplementary file 1 — Data S1. Supporting information. [file JFB-105-1562-s001.docx]

**Supporting information for**

**“****Size-selection drives genomic shifts in a harvested population.”**

Authors: Daniel E. Sadler, Tiina Sävilammi, Stephan van Dijk, Phillip C. Watts, Silva Uusi-Heikkilä

**Page Contents**

S2 Expanded Methodology

S3 Figure S1. Screeplot of the component-wise variance

S4 Figure S2: Functional categories of SNPs

S5 Figure S3: Diffstat differences

S6 Figure S4: F_st_ comparisons with simulated population

S7 Figure S5: Overlapping GOterms

S7 Table S1: Summary of replicates

S8 Table S2: Posthoc statistics of PC1

S8-S9 Table S3: Posthoc statistics of PC2

S9 Table S4: Summary statistics of F_st_

S10 Table S5: Summary of diversity results

**Methodology**

***SNP calling***

SNP calling resulted in 4,018,403 variable loci, of which we further excluded 589,356 multiallelic loci and 235 loci with other than alleles 0 or 1. The final data set consisted of 3,428,812 variable loci, and the assembled mean of 5.4x coverage.

***Kinship analysis***

To analyse differences in breeding coefficients, we used the program SEEKIN (Dou et al., 2017). Prior to using the SEEKIN software, we created vcf files subsetted by selection line using bcftools (v. 1.10 (using htslib 1.10)), then used kinship estimation for homogenous samples, with the following parameters: *-r 0.3 -m 0.05 -d GT -p hom -l 2000 -t 3 -w 1*.

**Polymorphism %**

We subsetted 100K random loci from the loci (including both variable and univariable loci) which were genotyped with <=30% missing samples. We then calculated the median fraction of observed polymorphic loci for each replicate, and non-parametric confidence intervals using 1000 bootstrap replicates with replacement.


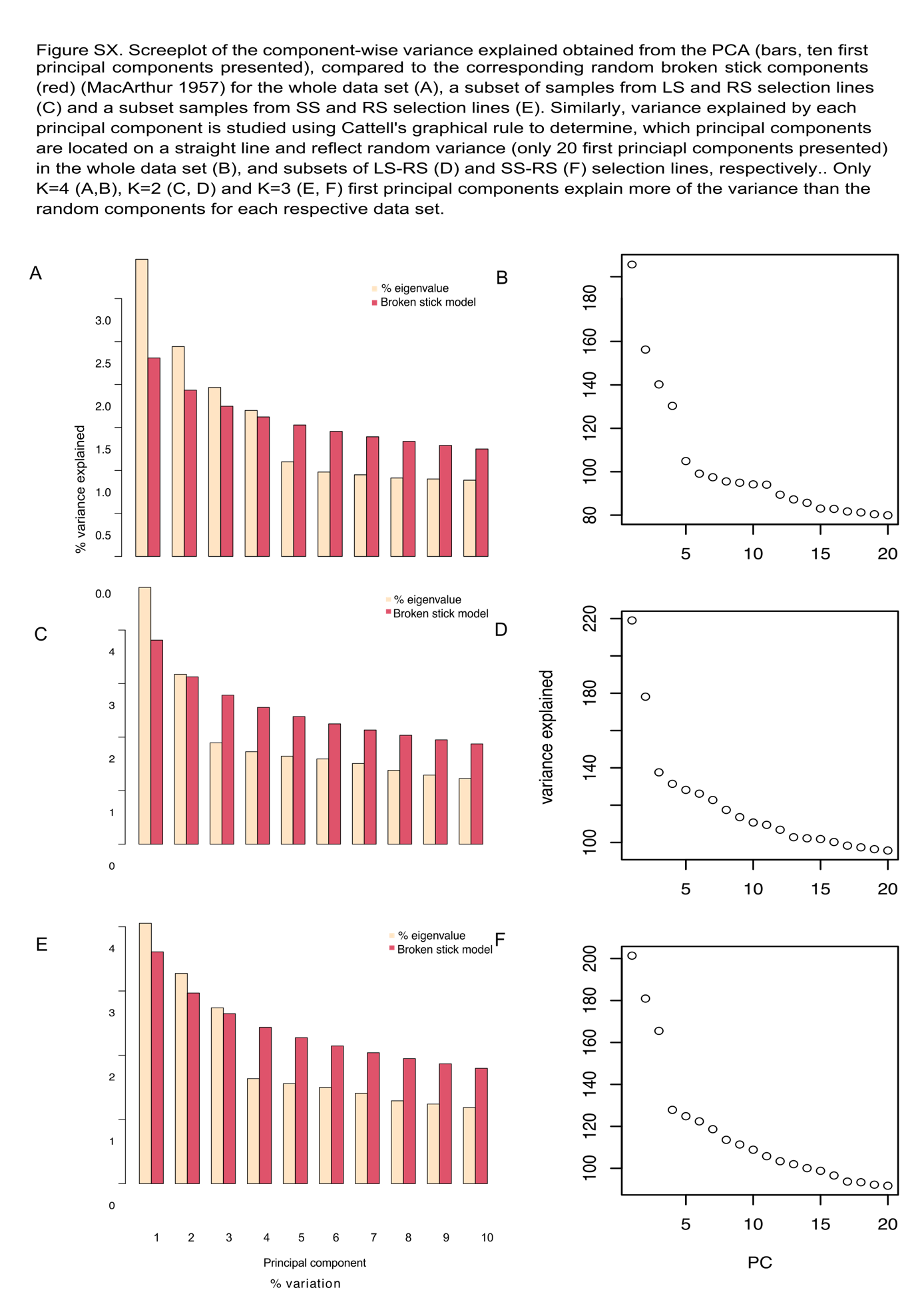


**Figure S1**. Screeplot of the component-wise variance explained obtained from the PCA (bars, ten first principal components presented), compared to the corresponding random broken stick components (red) for the whole data set (A), a subset of samples from LS and RS selection lines (C) and a subset samples from SS and RS selection lines (E). Similarly, variance explained by each principal component is studied using Cattell's graphical rule to determine, which principal components are located on a straight line and reflect random variance (only 20 first principal components presented) in the whole data set (B), and subsets of LS-RS (D) and SS-RS (F) selection lines, respectively. Only K=4 (A,B), K=2 (C, D) and K=3 (E, F) first principal components explain more of the variance than the random components for each respective data set.


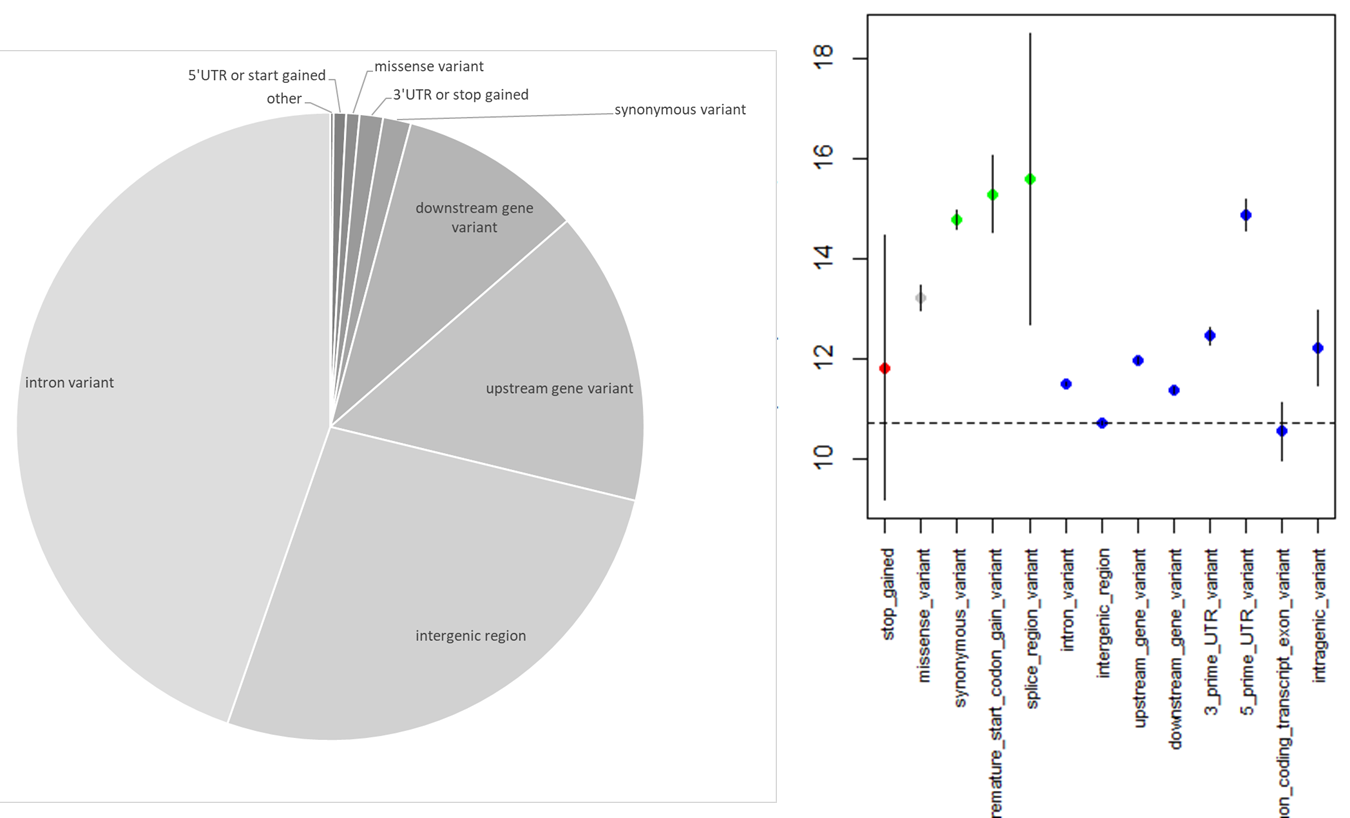


**Figure S2:** The potential functionalities of SNPs with respect to the affected genes (A). The mean first principal component loadings determined using PCAdapt analysis, with 95% confidence intervals (B). The colors represent the severity of the predicted effect to the resulting coding sequence; red, gray and blue represent high, intermediate and low severity, respectively; and blue represents the regulatory effect of the expression level. A horizontal line is drawn to highlight the mean value of SNPs in the intergenic regions (not associated with genes).

**
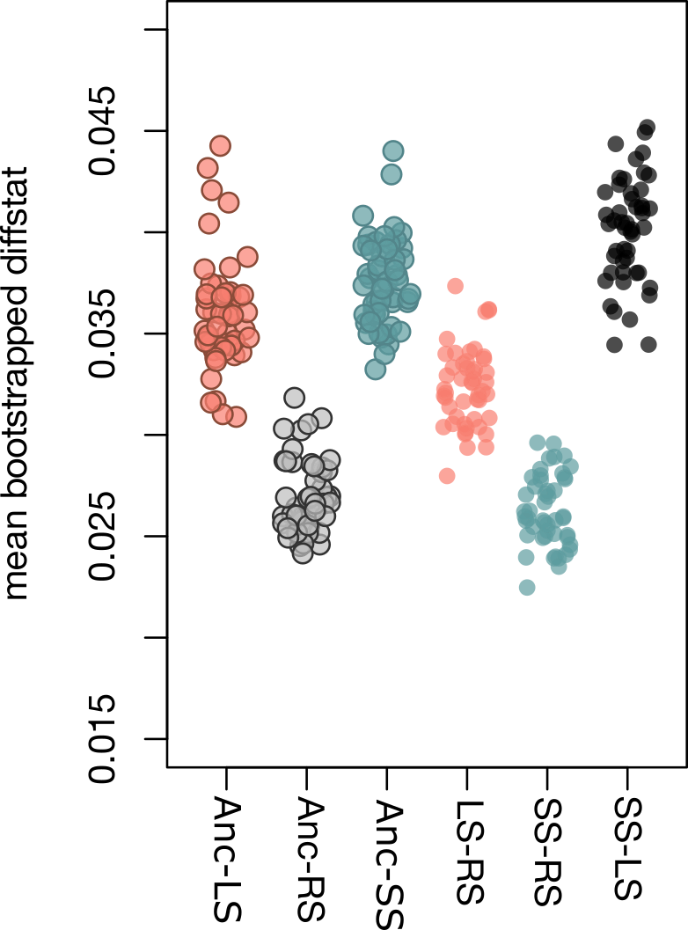
**

**Figure S3:** Mean bootsrapped diffstat between selection lines and between selection lines (LS, RS and SS) and founder population (ANC). Dots represent raw datapoints.


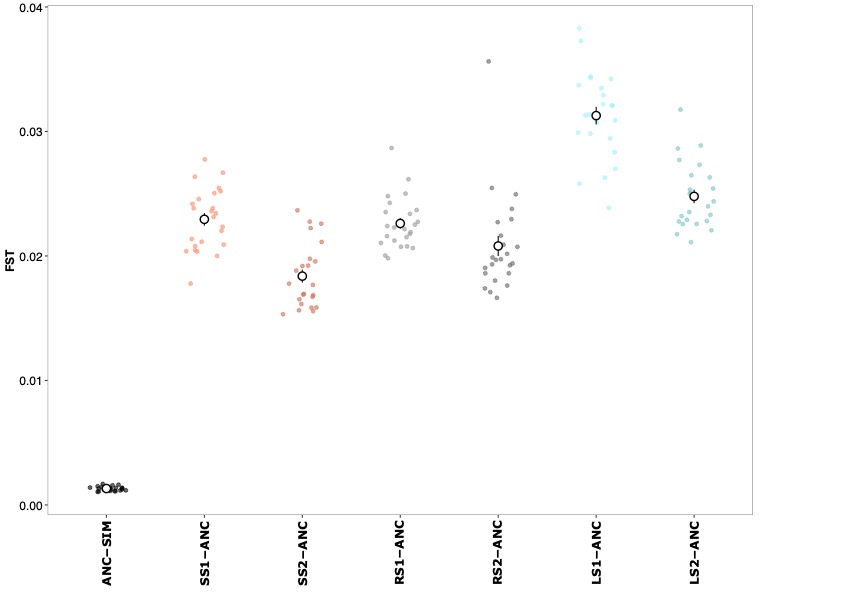


**Figure S4:** F_st_ across selection line replicates of the zebrafish: small selected (SS1; SS2), random selected (RS1; RS2), large selected (LS1; LS2) and the founder population (Anc). SIM is the simulated population F_st_ after six generations in absence of selection simulated using SLiM. Dots represent raw data, centroid represents mean of the data with SD lines.


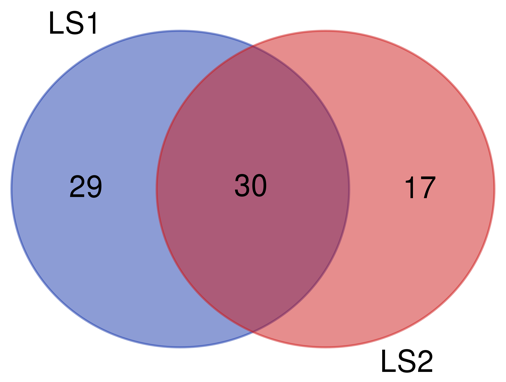

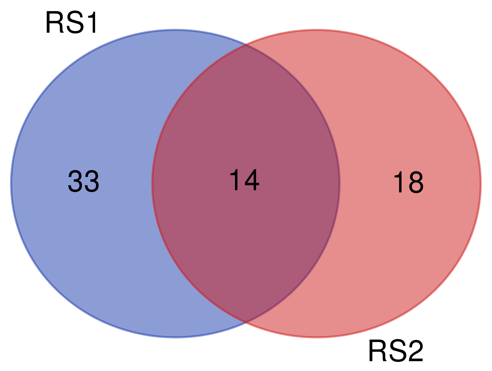

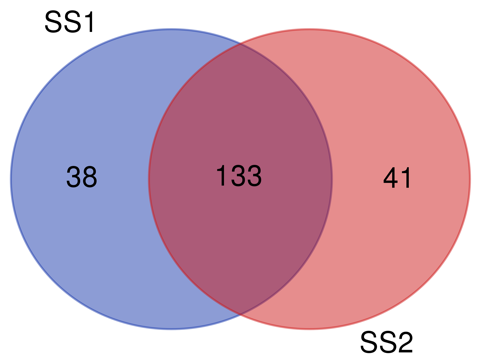

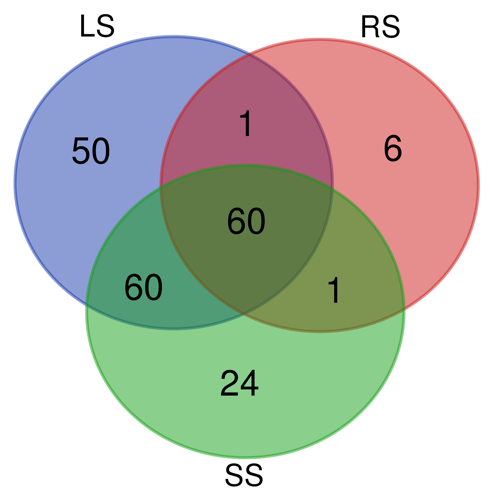


**Figure S5:** Overlapping GO terms for selection line replicates (a, b, c) and between each selection line replicate and founder population (d).

**Table S1:** Summary of selection line sample sizes.

|  | **Founder** | **SS1** | **SS2** | **RS1** | **RS2** | **LS1** | **LS2** |
| --- | --- | --- | --- | --- | --- | --- | --- |
| Samples | 42 | 41 | 28 | 43 | 34 | 39 | 40 |

**Table S2:** TukeyHSD posthoc summary statistics for selection line comparisons across PC1

| ***Line*** | ***diff*** | ***lwr*** | ***upr*** | ***p adj*** |  |
| --- | --- | --- | --- | --- | --- |
| *LS1-anc* | 0.07169404 | 0.05459961 | 0.08878846 | **0** | ******* |
| *LS2-anc* | 0.04257924 | 0.02358257 | 0.06157591 | **0** | ******* |
| *RS1-anc* | -0.0410561 | -0.0579482 | -0.0241641 | **0** | ******* |
| *RS2-anc* | -0.002184 | -0.0201469 | 0.01577879 | 0.9998217 |  |
| *SS1-anc* | -0.0998097 | -0.1171245 | -0.0824948 | **0** | ******* |
| *SS2-anc* | -0.0639092 | -0.0811115 | -0.046707 | **0** | ******* |
|  |  |  |  |  |  |
| *LS2-LS1* | -0.0291148 | -0.0482039 | -0.0100257 | 0.0001791 | ******* |
| *RS1-LS1* | -0.1127502 | -0.1297461 | -0.0957542 | **0** | ******* |
| *RS2-LS1* | -0.0738781 | -0.0919386 | -0.0558175 | **0** | ******* |
| *SS1-LS1* | -0.1715037 | -0.1889199 | -0.1540875 | **0** | ******* |
| *SS2-LS1* | -0.1356033 | -0.1529075 | -0.118299 | **0** | ******* |
| *RS1-LS2* | -0.0836354 | -0.1025435 | -0.0647273 | **0** | ******* |
| *RS2-LS2* | -0.0447633 | -0.0646338 | -0.0248927 | **0** | ******* |
| *SS1-LS2* | -0.1423889 | -0.1616756 | -0.1231022 | **0** | ******* |
| *SS2-LS2* | -0.1064885 | -0.1256742 | -0.0873028 | **0** | ******* |
| *RS2-RS1* | 0.03887209 | 0.02100295 | 0.05674124 | **0** | ******* |
| *SS1-RS1* | -0.0587535 | -0.0759711 | -0.0415359 | **0** | ******* |
| *SS2-RS1* | -0.0228531 | -0.0399575 | -0.0057488 | 0.0017805 | ******* |
| *SS1-RS2* | -0.0976256 | -0.1158949 | -0.0793563 | **0** | ******* |
| *SS2-RS2* | -0.0617252 | -0.0798878 | -0.0435626 | **0** | ******* |
| *SS2-SS1* | 0.03590041 | 0.01837842 | 0.05342239 | 0.0000001 | ******* |

**Table S3:** TukeyHSD posthoc summary statistics for selection line comparisons across PC1

| ***Line*** | ***diff*** | ***lwr*** | ***upr*** | ***p adj*** |  |
| --- | --- | --- | --- | --- | --- |
| *LS1-anc* | -0.0614979 | -0.0840923 | -0.0389034 | **0** | ******* |
| *LS2-anc* | -0.0388365 | -0.0639452 | -0.0137277 | **0.0001356** | ******* |
| *RS1-anc* | 0.07056985 | 0.04824287 | 0.09289683 | **0** | ******* |
| *RS2-anc* | 0.03425799 | 0.01051573 | 0.05800026 | **0.0005033** | ******* |
| *SS1-anc* | -0.0793126 | -0.1021983 | -0.0564269 | **0** | ******* |
| *SS2-anc* | -0.0347027 | -0.0574396 | -0.0119658 | **0.0001767** | ******* |
|  |  |  |  |  |  |
| *LS2-LS1* | 0.02266139 | -0.0025695 | 0.04789231 | 0.1104436 |  |
| *RS1-LS1* | 0.13206772 | 0.10960342 | 0.15453202 | **0** | ******* |
| *RS2-LS1* | 0.09575586 | 0.07188442 | 0.1196273 | **0** | ******* |
| *SS1-LS1* | -0.0178147 | -0.0408344 | 0.00520501 | 0.2481014 |  |
| *SS2-LS1* | 0.0267952 | 0.00392342 | 0.04966698 | **0.0103689** | ***** |
| *RS1-LS2* | 0.10940633 | 0.08441466 | 0.134398 | **0** | ******* |
| *RS2-LS2* | 0.07309447 | 0.04683072 | 0.09935822 | **0** | ******* |
| *SS1-LS2* | -0.0404761 | -0.0659682 | -0.014984 | **0.000079** | ******* |
| *SS2-LS2* | 0.0041338 | -0.0212248 | 0.02949238 | 0.999032 |  |
| *RS2-RS1* | -0.0363119 | -0.0599303 | -0.0126934 | **0.000153** | ******* |
| *SS1-RS1* | -0.1498824 | -0.1726397 | -0.1271252 | **0** | ******* |
| *SS2-RS1* | -0.1052725 | -0.1278801 | -0.0826649 | **0** | ******* |
| *SS1-RS2* | -0.1135706 | -0.1377179 | -0.0894233 | **0** | ******* |
| *SS2-RS2* | -0.0689607 | -0.092967 | -0.0449543 | **0** | ******* |
| *SS2-SS1* | 0.04460992 | 0.02145034 | 0.06776949 | **0.0000006** | ******* |

**Table S4:** Summary of F_st_ across selection lines and simulated lines of zebrafish.

| **Line** | **F_st_** | **se** | **ci** |
| --- | --- | --- | --- |
| **ANC-SIM** | 0.00131872 | 3.56E-05 | 7.37E-05 |
| **LS1-ANC** | 0.02294295 | 5.07E-04 | 1.05E-03 |
| **LS2-ANC** | 0.01838142 | 5.19E-04 | 1.07E-03 |
| **RS1-ANC** | 0.022612 | 4.23E-04 | 8.75E-04 |
| **RS2-ANC** | 0.02080235 | 8.08E-04 | 1.67E-03 |
| **SS1-ANC** | 0.03127603 | 7.03E-04 | 1.46E-03 |
| **SS2-ANC** | 0.02478547 | 5.38E-04 | 1.11E-03 |

**Table S5**: Diversity measures across selection line replicates

|  | | **Anc** | **SS1** | **SS2** | **RS1** | **RS2** | **LS1** | **LS2** |
| --- | --- | --- | --- | --- | --- | --- | --- | --- |
| **π** | mean | 0.000511 | 0.000606 | 0.000514 | 0.000536 | 0.000481 | 0.000545 | 0.000518 |
|  | CI | 0.000051 | 0.000010 | 0.0000008 | 0.0000029 | 0.0000025 | 0.0000039 | 0.0000012 |
| **H_exp_** | mean | 0.246887 | 0.246431 | 0.246335 | 0.246687 | 0.246509 | 0.246236 | 0.246417 |
|  | CI | 0.000268 | 0.000246 | 0.000378 | 0.000312 | 0.000277 | 0.000318 | 0.000248 |
| ***Ne*** | mean | 120.7 | 38 | 68 | 43.8 | 70.5 | \| 52.7 \| \| --- \| \|  \| | 64.1 |
|  | CI | 46.6 | 19.3 | 23.5 | 11.6 | 13.7 | 21.8 | 17.2 |
| **Polymorph-ism**  **(%)** | mean | 11.3 | 9.7 | 8.9 | 9.7 | 9.2 | 9.0 | 9.6 |
|  | CI | 0.2 | 0.2 | 0.1 | 0.1 | 0.2 | 0.2 | 0.2 |
